# Supplementary material for: Long-read cDNA sequencing identifies functional pseudogenes in the human transcriptome
Source: Genome Biol. 2021 May 10;22:146. doi: 10.1186/s13059-021-02369-0 (PMC8108447; doi:10.1186/s13059-021-02369-0)
Supplement: Supplementary file 1 — Additional file 1. Supplementary figures. [file 13059_2021_2369_MOESM1_ESM.pdf]

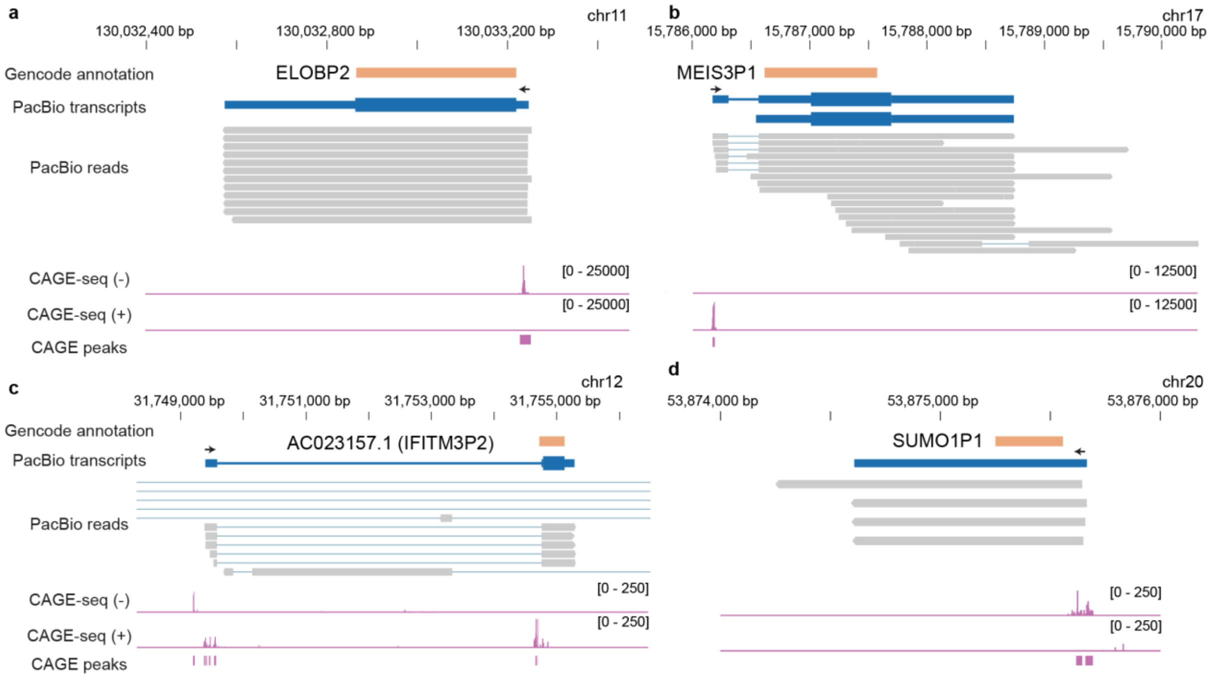

Fig. S1 a PacBio Iso-Seq reads identify the full-length ELOBP2 transcript. The TSS of ELOBP2 is supported by a CAGE peak. **b** PacBio Iso-Seq reads identify the full-length MEIS3P1 transcript. The TSS of MEIS3P1 is supported by a CAGE peak. **c** PacBio Iso-Seq reads identify the full-length IFITM3P2 transcript. The TSS of IFITM3P2 is supported by a CAGE peak. **d** PacBio Iso-Seq reads identify the full-length SUMO1P1 transcript. The TSS of SUMO1P1 is supported by a CAGE peak.

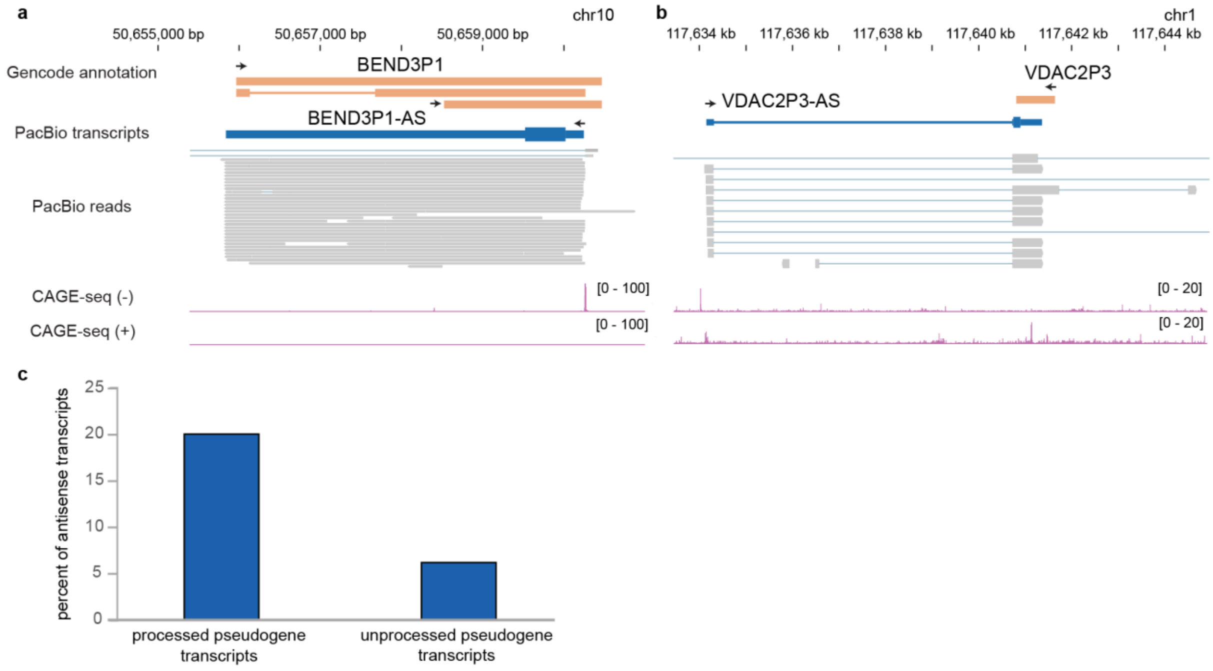

Fig. S2 a BEND3P1 is expressed in the antisense orientation with respect to its parent gene. **b** The spliced pseudogene transcript VDAC2P3 is expressed in the antisense orientation with respect to its parent gene. **c** A higher fraction of processed pseudogenes transcripts are antisense compared to unprocessed pseudogenes.

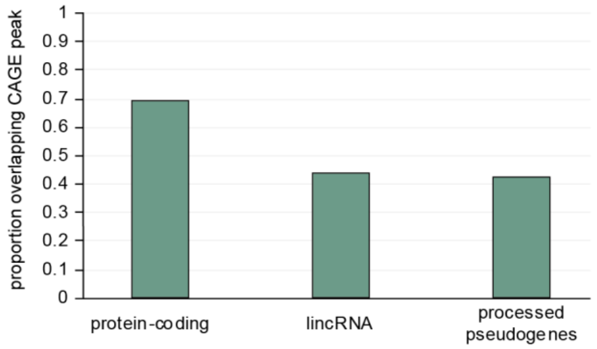

Fig. S3 Proportion of transcripts of different biotypes overlapping with FANTOM5 CAGE peaks.

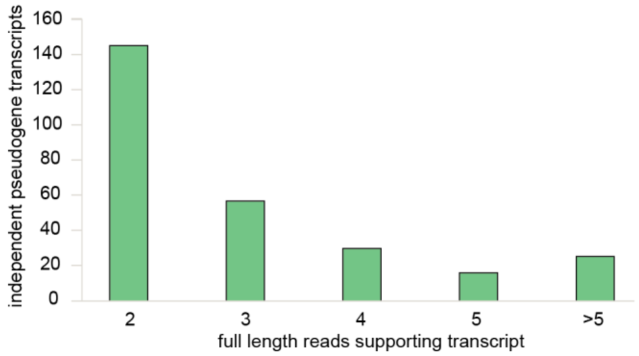

Fig. S4 Distribution of number of full-length reads supporting each pseudogene transcript model. The minimum required reads is two.

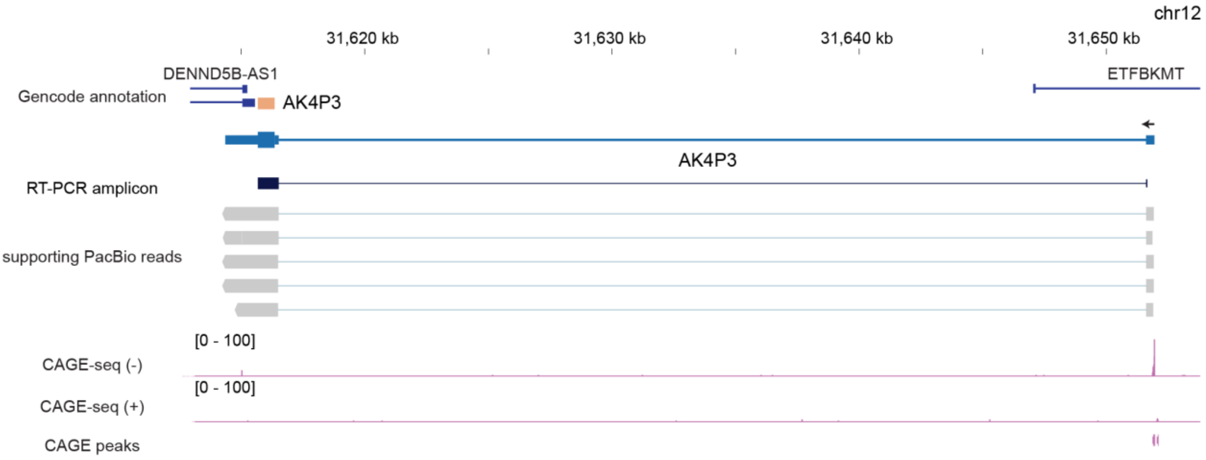

Fig. S5 AK4P3 a transcribed potentially-coding pseudogene. AK4P3 has a novel 5' exon and is transcribed from an upstream CAGE-confirmed TSS.

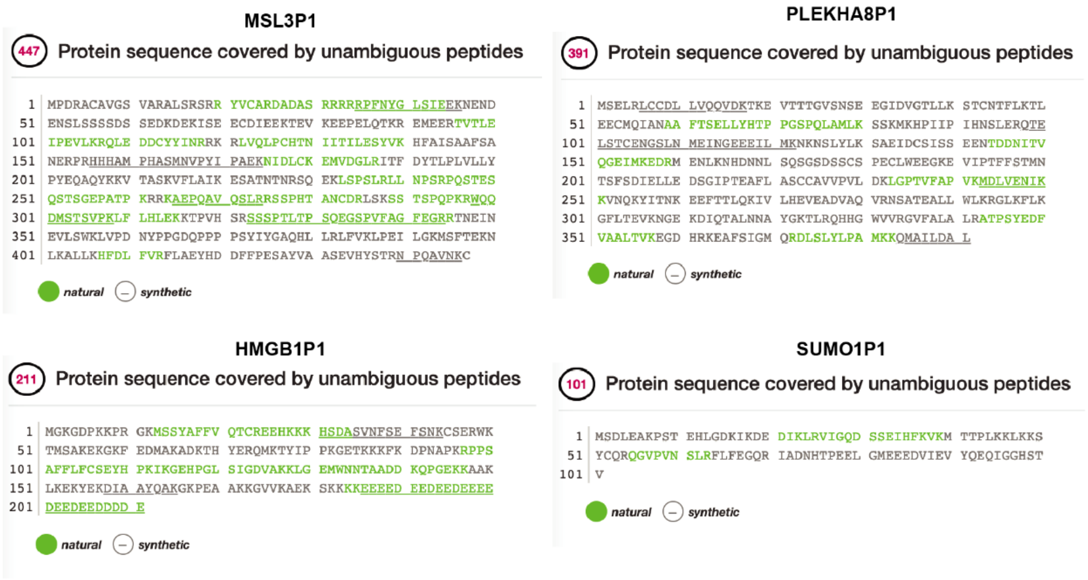

Fig. S6 neXtprot peptide coverage of four translated pseudogenes. Natural peptides are those observed in mass-spectrometry experiments, whilst synthetic peptides are artificial standards.

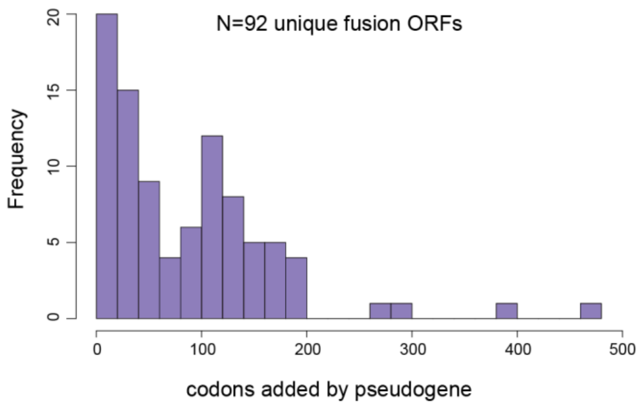

Fig. S7 Distribution of the number of codons that pseudogenes contribute to known protein-coding genes.

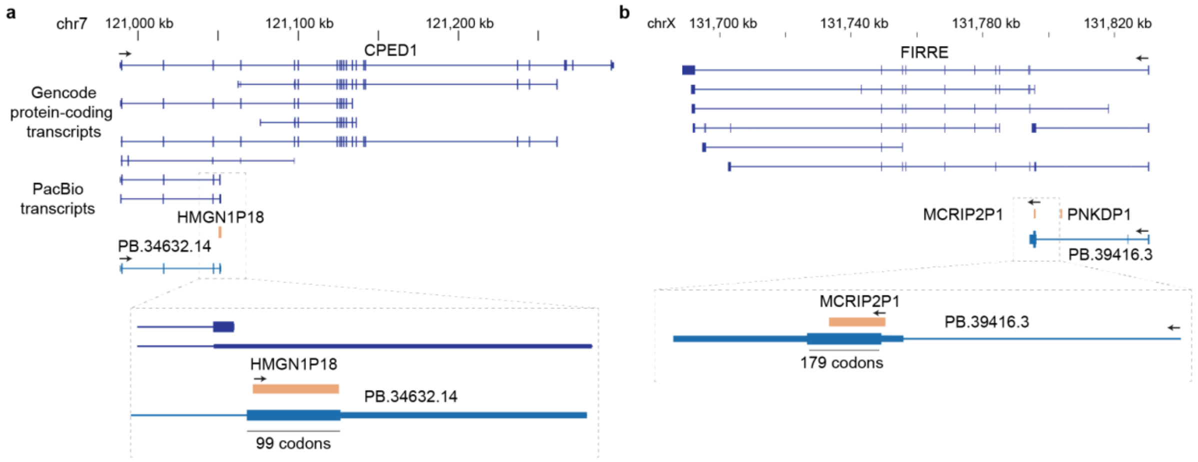

Fig. S8 a A novel CPED1 isoform contains a HMGN domain encoded by the pseudogene HMGN1P18. **b** An isoform of the lncRNA FIRRE splices into MCRIP2P1 and may encode a protein.

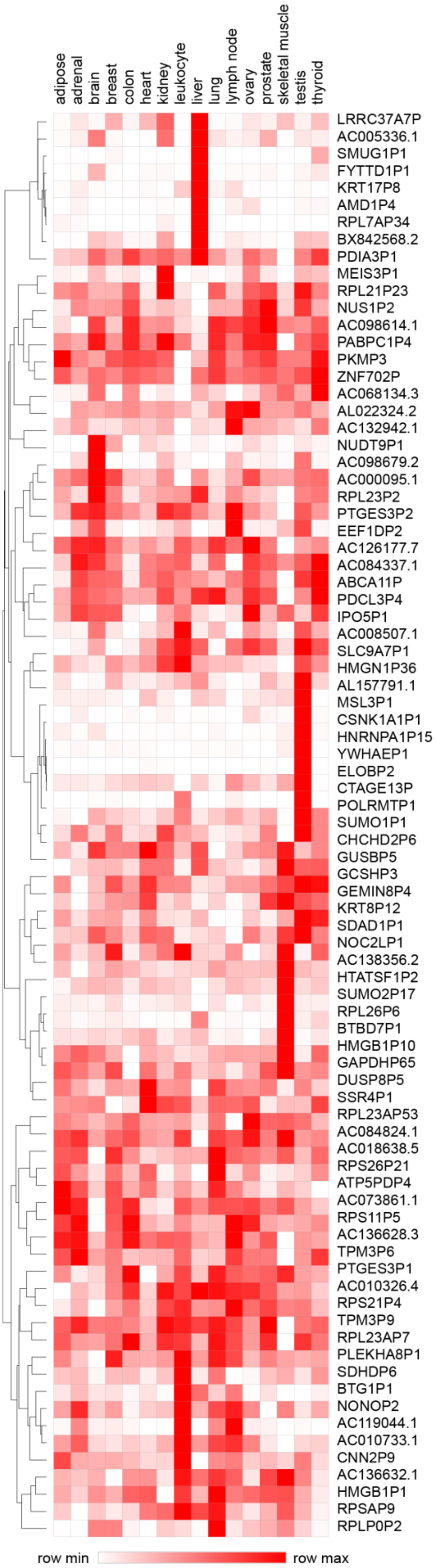
adipose
adrenal
brain
breast
colon
heart
kidney
leukocyte
liver
lung
lymph node
ovary
prostate
skeletal muscle
testis
thyroid
LRRC37A7P
AC005336.1
SMUG1P1
FYTTD1P1
KRT17P8
AMD1P4
RPL7AP34
BX842568.2
PDIA3P1
MEIS3P1
RPL21P23
NUS1P2
AC098614.1
PABPC1P4
PKMP3
ZNF702P
AC068134.3
AL022324.2
AC132942.1
NUDT9P1
AC098679.2
AC000095.1
RPL23P2
PTGES3P2
EEF1DP2
AC126177.7
AC084337.1
ABCA11P
PDCL3P4
IPO5P1
AC008507.1
SLC9A7P1
HMGN1P36
AL157791.1
MSL3P1
CSNK1A1P1
HNRNPA1P15
YWHAEP1
ELOBP2
CTAGE13P
POLRMTP1
SUMO1P1
CHCHD2P6
GUSBP5
GCSHP3
GEMIN8P4
KRT8P12
SDAD1P1
NOC2LP1
AC138356.2
HTATSF1P2
SUMO2P17
RPL26P6
BTBD7P1
HMGB1P10
GAPDHP65
DUSP8P5
SSR4P1
RPL23AP53
AC084824.1
AC018638.5
RPS26P21
ATP5PDP4
AC073861.1
RPS11P5
AC136628.3
TPM3P6
PTGES3P1
AC010326.4
RPS21P4
TPM3P9
RPL23AP7
PLEKHA8P1
SDHDP6
BTG1P1
NONOP2
AC119044.1
AC010733.1
CNN2P9
AC136632.1
HMGB1P1
RPSAP9
RPLP0P2
row min
row max

Fig. S9 Tissue-specific pseudogene expression in the 16 adult tissues of the Illumina Body Map. Heatmap was generated using Morpheus, https://software.broadinstitute.org/morpheus. Expression is represented as log2(cpm+1) and is row scaled. Only sense independent pseudogenes with expression of greater than one cpm in at least one tissue are shown.

Supplementary figure 10

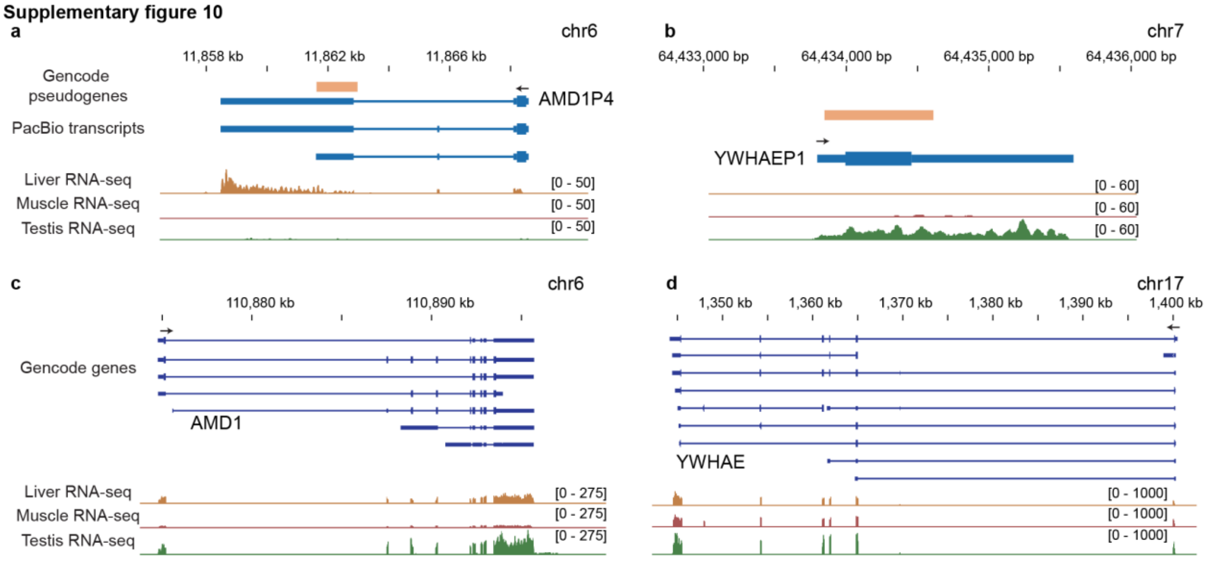

Fig. S10 a AMD1P4 is expressed exclusively in the liver. **b** YWHAEP1 is expressed exclusively in the testis. **c** AMD1 is expressed broadly. **d** YWHAE is expressed broadly.

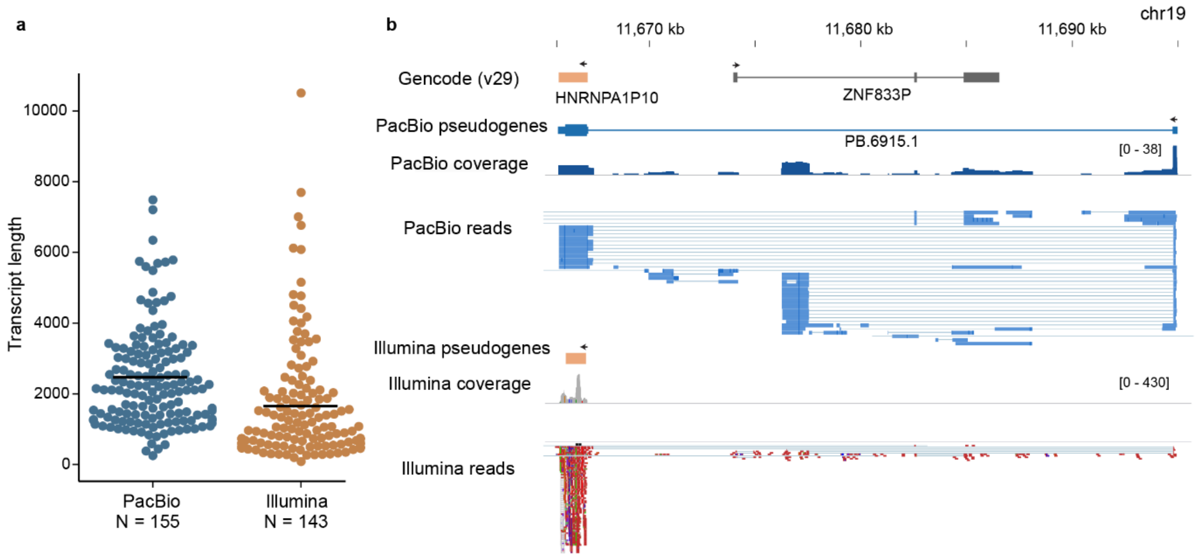

Fig. S11 a Average of length of pseudogene transcripts assembled by StringTie [30] from Illumina data compared to PacBio pseudogene transcripts. Plot generated with Estimation Stats [1] **b** PacBio sequencing identifies a HNRNPA1P10 isoform that is not detected by short-read assembly.

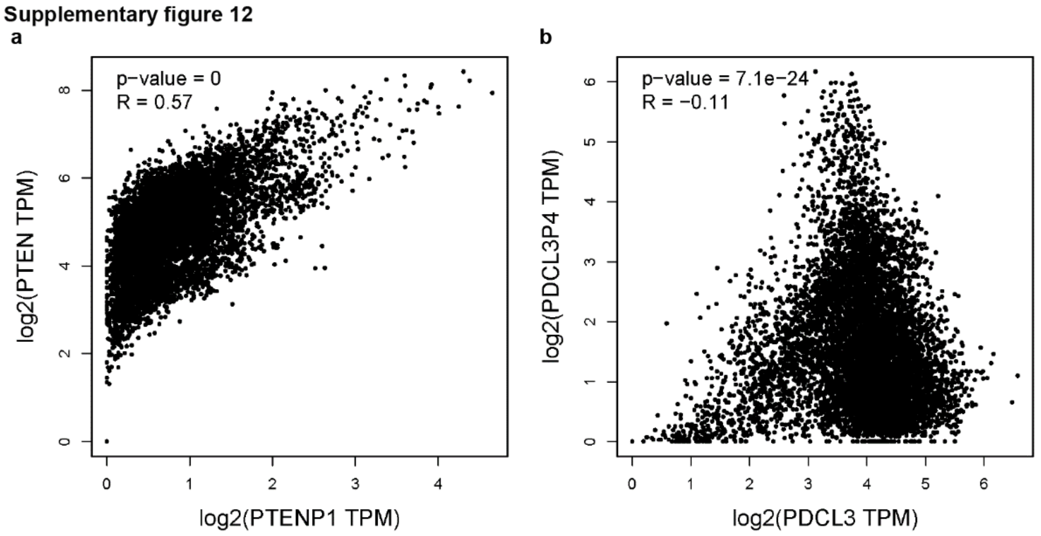

Fig. S12 a PTEN and PTENP1 are strongly correlated in GTEX tissues. Correlation plots were generated with GEPIA [2]. **b** PDCL3P4 and PDCL3 expression is not positively correlated in GTEX [3] tissues.

References

1. Ho J, Tumkaya T, Aryal S, Choi H, Claridge-Chang A. Moving beyond P values: data analysis with estimation graphics. Nat Methods. 2019;16:565–6.

2. Tang Z, Li C, Kang B, Gao G, Li C, Zhang Z. GEPIA: a web server for cancer and normal gene expression profiling and interactive analyses. Nucleic Acids Res. 2017;45:W98–102.

3. Lonsdale J, Thomas J, Salvatore M, Phillips R, Lo E, Shad S, et al. The Genotype-Tissue Expression (GTEx) project. Nat Genet. 2013;45:580–5.
