# Supplementary figures and images for: Long-read cDNA sequencing identifies functional pseudogenes in the human transcriptome
Source: Genome Biol. 2021 May 10;22:146. doi: 10.1186/s13059-021-02369-0 (PMC8108447; doi:10.1186/s13059-021-02369-0)

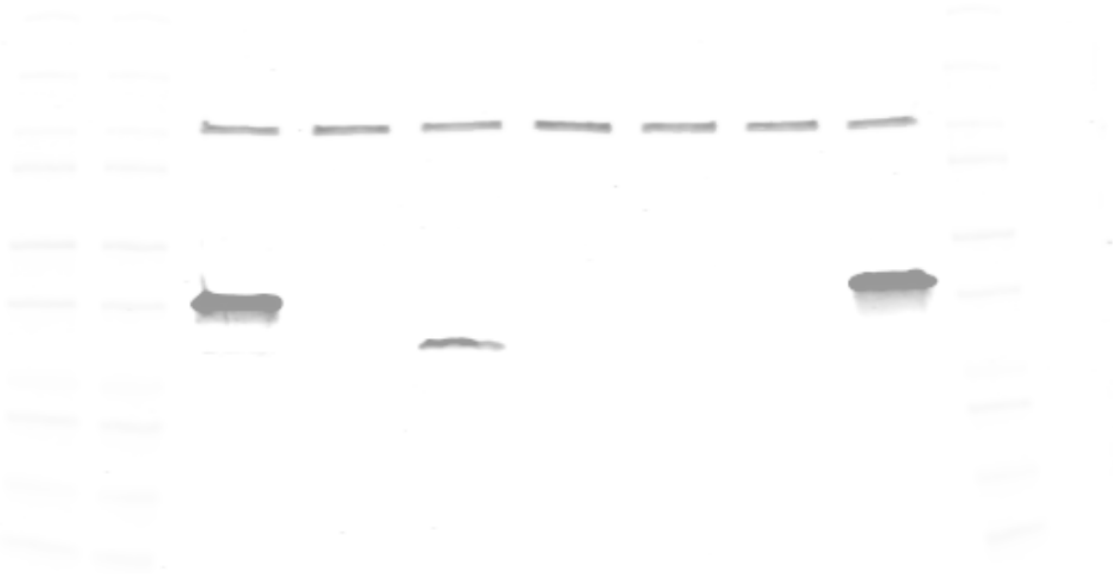

Additional file 4: Uncropped Western blot from Fig. 1g

Supplement: Supplementary file 4 — Additional file 4. Uncropped Western blot from Fig. 1g. [file 13059_2021_2369_MOESM4_ESM.pdf]
