# Supplementary material for: Long-read cDNA sequencing identifies functional pseudogenes in the human transcriptome
Source: Genome Biol. 2021 May 10;22:146. doi: 10.1186/s13059-021-02369-0 (PMC8108447; doi:10.1186/s13059-021-02369-0)
Supplement: Supplementary file 11 — Additional file 11. Review history. [file 13059_2021_2369_MOESM11_ESM.docx]

Review History

**First round of review**

**Reviewer 1**

**Are you able to assess all statistics in the manuscript, including the appropriateness of statistical tests used?**

Yes, and I have assessed the statistics in my report.

**Comments to author:**

Troskie et al. used long-read RNA-seq to detect expressions of human "pseudogenes". Due to the power of the long-read RNA-seq, they detected previously undetected expressed pseudogenes. They provided several lines of evidence for the biochemical activities of a few of these expressed pseudogenes, such as their translation and impact on the expressions of other genes when deleted. The work is straightforward and the writing is generally clear. I have one major comment and a suggestion.

1. As I am sure the authors are aware (based on their review in Nat Rev Genet), the functionality of a gene or pseudogene is proven only by showing that the (pseudo)gene is subject to selection. Other activities such as binding to other molecules, translation, and regulating other genes can be regarded as biochemical activities, which may or may not form biological functions. Thus, to claim that the pseudogenes they identified are functional, they should provide evidence for selection. This is the standard for claiming functional pseudogenes (e.g., PMID: 26589994).

2. Because CRISPR-Cas9 can create off-target mutations, it is important to demonstrate that the effect on the expressions of other genes that they observed in KO lines is not due to off-target mutations. Fortunately, the authors have several KO lines. Based on the results presented in Table S3, I can tell that most the effects are genuine (because they are shared among KO lines). I suggest that the authors mention this in the main text to strengthen their argument.

**Reviewer 2**

**Are you able to assess all statistics in the manuscript, including the appropriateness of statistical tests used?**

Yes, and I have assessed the statistics in my report.

**Comments to author:**

Troskie and colleagues describe the use of long read sequencing technologies to catalogue transcribed pseudogenes in human cells and tissues. The authors carried out a survey of a subset of properties of these transcribed pseudogenes and investigate the impact of the loss of function of one pseudogene on gene expression. Whereas the present catalogue is of potential interest to the genomics community, some aspects of the analysis are superficial and overall the conclusions are descriptive and not novel.

Specific comments:

1-Others have used short read data to characterize pseudogene transcription. A direct comparison between data from the two sources may allow to assess the added value and the limitations of the present catalogue.

2- The authors use cage to further support that the transcript models of of the identified pseudogenes. They find that only 36% had a cage tag within 100 bp of the predicted TSS. Does this mean that the remaining 63% have no cage? How does this compare to other gene classes.

3-It is unclear to this reviewer how the cage data analysis that supports the expression of only a minority of the pseudogenes (36%) indicates that "data still significantly underestimates pseudogene transcription.

4-22% of the pseudogenes reported here are transcribed in the antisense direction. How does this compare between pseudogenes with different origin and is this more than one would expect suggesting that indeed this type of transcription is retained because it has the potential to regulate the parental gene expression? Is there a preference for where in the pseudogene sequence does transcription starts?

5-It has been previously described that genes annotated as "pseudogenes" retain coding potential and can add protein domains to other genes. The added value of the present coding potential analysis is unclear. Furthermore, presence of an ORF per se is not sufficient to support translation.

6- The analysis of PDCL3P4 is superficial. The absence of a rescue experiment and at least preliminary insights on the mechanism make the present results correlative.

**Reviewer 3**

**Are you able to assess all statistics in the manuscript, including the appropriateness of statistical tests used?**

Yes, and I have assessed the statistics in my report.

**Comments to author:**

In this article the authors have presented how PacBio full-length cDNA sequencing of 20 normal tissues and 10 cancer cell lines has enabled the identification of 199 novel transcribed pseudogenes not present in the GENCODE reference. They have conducted comprehensive bioinformatics analysis of the transcribed pseudogenes looking at expression levels and CAGE tags to indicate TSSs sites. They have also conducted biochemical analysis investigating the regulatory PDCL3P4 in HAP1 cells and finding it disrupts many genes but not the parent gene PDCL3 expression and could be acting as an lncRNA. In general, I think this is a comprehensive analysis showing the advantage of using long read technology to identify transcribed pseudogenes. However, I would like the authors to clarify the following points:

1. The reference Gou et al highlighted 1,326 transcribed pseudogenes in GENCODE in 2014 and currently in the GENCODE release 36 there are 939 transcribed unprocessed pseudogenes and 500 processed pseudogenes. Therefore why is the total number of GENCODE pseudogenes quoted as 375 in Fig1 b, as this doesn't seem to correspond and I would expect more overlap with the PacBio transcribed pseudogenes.

2. Probably the most contentious issue around transcribed pseudogenes is how many could actually code for proteins, since the majority have found to have a regulatory effectas a lncRNA. The authors mention 93 expressed pseudogenes have protein coding potential and highlight CAGE data indicating that they are full length transcripts. Yet there is no mention of identifying polyA sites and signals, which would influence the transcripts stability and if it would indeed produce a viable protein coding transcript invivo. Can the authors comment on if they have done this analysis and how many of their transcribed pseudogenes have polyA features. Also have the authors also considered using a proteogenomic workflow as described by Wright et al Nat comms 2016 to identify if mass spec data can support translation of the potential new translated pseudogenes invivo?

3. The FIRRE transcript appears to have two processed pseudogenes overlapping the transcripts annotated in ENSEMBL MRCIP2P1 and also PNKDP1. Does the 2nd pseudogene PNKDP1 also have a spliced isoform and should it be added to Fig S6b?

4. The authors mention the expression levels of the expressed pseudogenes being different than their parent genes but in Fig S8a-b the expression profile of the pseudogenes across the large exons are not uniform compared with the parent exons and is this due to potential mis- mapping of the short illumina reads?

**Authors Response**

**Point-by-point responses to the reviewers’ comments:**

Reviewer #1: Troskie et al. used long-read RNA-seq to detect expressions of human "pseudogenes". Due to the power of the long-read RNA-seq, they detected previously undetected expressed pseudogenes. They provided several lines of evidence for the biochemical activities of a few of these expressed pseudogenes, such as their translation and impact on the expressions of other genes when deleted. The work is straightforward and the writing is generally clear. I have one major comment and a suggestion.

1. As I am sure the authors are aware (based on their review in Nat Rev Genet), the functionality of a gene or pseudogene is proven only by showing that the (pseudo)gene is subject to selection. Other activities such as binding to other molecules, translation, and regulating other genes can be regarded as biochemical activities, which may or may not form biological functions. Thus, to claim that the pseudogenes they identified are functional, they should provide evidence for selection. This is the standard for claiming functional pseudogenes (e.g., PMID: 26589994).

*We strongly agreed with this point and therefore determined the extent of selection on conserved pseudogene ORFs, finding that most show evidence. We have added the following text to page 5 paragraph 2 and a new supplementary table (S2) outlining these results.*

*“To determine if the pseudogene ORFs are subject to purifying selection we identified orthologous positions in non-human primate genomes by aligning the human transcripts with a 1000bp window on each side to higher primates (chimp, gorilla, orangutan, and rhesus). The alignments were then further refined to identify orthologous cDNA sequences and the resulting ORFs were translated into amino acid sequences (Methods). The extent of selection on pseudogene ORFs was determined by maximum likelihood estimation of the ratio of substitution rates between two divergent species that result in nonsynonymous vs synonymous changes (dN/dS). A ratio of >1 suggests diversifying selection whilst a ratio of <1 is consistent with purifying selection. This index has been used as evidence of conserved function for human processed pseudogenes (Xu and Zhang 2016). Of the 32 pseudogene ORFs which are conserved in Rhesus Macaque and have sufficient nucleotide diversity 29/35 (85%) have a dN/dS <1 (median 0.4483) suggesting that most of the conserved pseudogene ORFs were under purifying selection across 25M years of evolution (Table S2).”*

2. Because CRISPR-Cas9 can create off-target mutations, it is important to demonstrate that the effect on the expressions of other genes that they observed in KO lines is not due to off-target mutations. Fortunately, the authors have several KO lines. Based on the results presented in Table S3, I can tell that most of the effects are genuine (because they are shared among KO lines). I suggest that the authors mention this in the main text to strengthen their argument.

*We thank Reviewer #1 for pointing this out. We agree and have added the following text to page 8 paragraph 1 to strengthen this argument:*

*“Changes in gene expression were consistent between the independent clonal knockouts, indicating that these expression changes do not represent off-targets.”*

Reviewer #2: Troskie and colleagues describe the use of long read sequencing technologies to catalogue transcribed pseudogenes in human cells and tissues. The authors carried out a survey of a subset of properties of these transcribed pseudogenes and investigated the impact of the loss of function of one pseudogene on gene expression. Whereas the present catalogue is of potential interest to the genomics community, some aspects of the analysis are superficial and overall the conclusions are descriptive and not novel.

Specific comments:

1-Others have used short read data to characterize pseudogene transcription. A direct comparison between data from the two sources may allow to assess the added value and the limitations of the present catalogue.

*This suggestion had significant merit. To compare short-read and long-read approaches to survey pseudogene transcription, we performed short-read assembly using StringTie and compared the transcripts to those identified by our PacBio pipeline. We found that StringTie does not detect all PacBio-assembled pseudogene transcripts. Those transcripts that are identified by StringTie are incomplete, demonstrating the value of our long-read approach. We have added a supplementary figure and text to describe this analysis on page 7 paragraph 2:*

*“To determine if long-read sequencing outperforms short-read sequencing at assembling pseudogene transcripts we generated matched PacBio and Illumina datasets from the haploid leukaemia cell line HAP1. Without a reference transcriptome, 71% of the 163 HAP1 PacBio pseudogene transcripts were detected by short-read transcript assembly with StringTie (Pertea et al. 2015), whilst with Gencode (v29) as a reference 91% of pseudogene transcripts were detected. However, the reference-guided short-read assemblies were significantly shorter than the PacBio transcripts (average of 750bp shorter, p=8.5E-11 Mann-Whitney) indicating that these assemblies do not cover complete transcripts (Fig. S11a-b).”*

2- The authors use cage to further support the transcript models of the identified pseudogenes. They find that only 36% had a cage tag within 100 bp of the predicted TSS. Does this mean that the remaining 63% have no cage? How does this compare to other gene classes.

*To address this point, we performed an additional analysis and found that 69% of protein-coding transcript TSSs, and 44% of lincRNA transcript TSSs, overlap with at least one CAGE peak. This analysis is presented in the new Fig. S3 and the following text was added to page 4 paragraph 2:*

*“The proportion of pseudogene transcripts that overlap CAGE peaks is lower than for protein-coding transcripts (69%) and comparable to lincRNA transcripts (44%) (Fig. S3).”*

3-It is unclear to this reviewer how the cage data analysis that supports the expression of only a minority of the pseudogenes (36%) indicates that "data still significantly underestimates pseudogene transcription.

*CAGE-seq reads are short and have limited mappability within pseudogenes. Additionally, pseudogenes have lower expression than protein-coding genes and can be highly-tissue specific. We have elaborated this point in new text on page 4 paragraph 2.*

*“51% of independent pseudogene transcripts were supported by the cutoff of two full-length reads (Fig. S4), and our datasets do not comprehensively capture diversity of human cell-types and developmental stages. Therefore it is probable that our data still significantly underestimate pseudogene transcription; further transcripts would very likely be identified by increased sequencing depth applied to individual tissues or cell types.”*

4-22% of the pseudogenes reported here are transcribed in the antisense direction. How does this compare between pseudogenes with different origin and is this more than one would expect suggesting that indeed this type of transcription is retained because it has the potential to regulate the parental gene expression? Is there a preference for where in the pseudogene sequence does transcription start?

*These are good points and led us to re-examine our data. We have added the following text to page 4 paragraph 2 and added a supplementary figure (Fig. S2c).*

*“In contrast, only 68/2669 (6.3%) of unprocessed pseudogenes are transcribed in antisense (Fig S2c). This difference may be attributable either to the propensity for novel downstream promoter elements to regulate expression of a retrotransposed pseudogene relative to unprocessed pseudogenes (which retain their parental promoter) or to selection for regulatory potential. Manual inspection of the antisense pseudogene TSSs did not reveal an obvious initiation site bias.”*

5-It has been previously described that genes annotated as "pseudogenes" retain coding potential and can add protein domains to other genes. The added value of the present coding potential analysis is unclear. Furthermore, presence of an ORF per se is not sufficient to support translation.

*We agree that ORF annotation is not sufficient to provide evidence for translation. In the initial submission, we tested whether a subset of pseudogene transcripts could be translated in human cells (Fig. 1g). In the revised submission, we have now interrogated a large mass-spectrometry database (neXtprot). Eleven of the potentially coding pseudogenes are included in the database as putative proteins. Four of these have extensive coverage with peptides from mass-spectrometry experiments, confirming that annotated pseudogene transcripts can encode proteins. We have added Fig. S6 and the following text on page 5 paragraph 2:*

*“To further substantiate that pseudogenes can be translated in vivo we interrogated the neXtprot human proteomics database (Zahn-Zabal et al. 2020). Eleven potentially-coding pseudogenes have entries in neXtprot of which four, HMGB1P1, SUMO1P1, MSL3P1 and PLEKHA8P1 have matched unique peptides (Fig. S6).”*

6- The analysis of PDCL3P4 is superficial. The absence of a rescue experiment and at least preliminary insights on the mechanism make the present results correlative.

*While we agree that more experimental data regarding the function of PDCL3P4 would be desirable in the future, the reasonable timeframe of revision would make such experiments at best superficial and at worst prone to erroneous conclusions. We have added the following line to the conclusion on page 8 paragraph 2 emphasising that the mechanism of action remains to be determined:*

*“Future work will elucidate the mechanism through which PDCL3P4 affects gene expression.”*

Reviewer #3:

In this article the authors have presented how PacBio full-length cDNA sequencing of 20 normal tissues and 10 cancer cell lines has enabled the identification of 199 novel transcribed pseudogenes not present in the GENCODE reference. They have conducted comprehensive bioinformatics analysis of the transcribed pseudogenes looking at expression levels and CAGE tags to indicate TSSs sites. They have also conducted biochemical analysis investigating the regulatory PDCL3P4 in HAP1 cells and finding it disrupts many genes but not the parent gene PDCL3 expression and could be acting as an lncRNA. In general, I think this is a comprehensive analysis showing the advantage of using long read technology to identify transcribed pseudogenes. However, I would like the authors to clarify the following points:

1. The reference Gou et al highlighted 1,326 transcribed pseudogenes in GENCODE in 2014 and currently in the GENCODE release 36 there are 939 transcribed unprocessed pseudogenes and 500 processed pseudogenes. Therefore why is the total number of GENCODE pseudogenes quoted as 375 in Fig1 b, as this doesn't seem to correspond and I would expect more overlap with the PacBio transcribed pseudogenes.

*The previous version of the manuscript mistakenly listed the number of transcribed processed pseudogenes in Gencode(v29) as 375. We apologise and have corrected this to 481, of which 43 overlap with our processed pseudogene transcript models. Our processed pseudogene models number has increased due to the improper exclusion in the original manuscript of some models that are intronic to other processed pseudogene transcripts. We thank Reviewer #3 for bringing this issue to our attention.*

2. Probably the most contentious issue around transcribed pseudogenes is how many could actually code for proteins, since the majority have found to have a regulatory effectas a lncRNA. The authors mention 93 expressed pseudogenes have protein coding potential and highlight CAGE data indicating that they are full length transcripts. Yet there is no mention of identifying polyA sites and signals, which would influence the transcripts stability and if it would indeed produce a viable protein coding transcript invivo. Can the authors comment on if they have done this analysis and how many of their transcribed pseudogenes have polyA features.

Also have the authors also considered using a proteogenomic workflow as described by Wright et al Nat comms 2016 to identify if mass spec data can support translation of the potential new translated pseudogenes invivo?

*With regards to polyadenylation, we agree this feature is very important to evaluate the protein-coding potential of pseudogene RNAs. Fortunately, all of the pseudogene transcripts examined in this study are polyadenylated; the PacBio Iso-Seq protocol primes from the polyA tail using an oligodT primer and the analysis pipeline requires a polyA tail to include transcripts. We have added text noting the pseudogene transcripts are polyadenylated on page 4 paragraph 2.*

*“We identified 1170 polyadenylated transcripts, each supported by at least two full-length reads, that overlapped 521 processed pseudogenes”*

*We also added a line to emphasise the polyA+ve library preparation in the methods on page 9 paragraph 2.*

*“Libraries were prepared using the PacBio Iso-Seq library preparation, which amplifies full-length polyadenylated transcripts without size selection.”*

*With regards to further evidence of pseudogene translation, we agreed this would be highly desirable and therefore searched a public mass-spectrometry database (neXtprot) for potential pseudogene derived peptides. Eleven of the putatively translated pseudogenes described here had entries, of which four had extensive coverage of unambiguous mass-spectrometry peptides. To present these data, we have added Fig. S6 and the following text on page 5 paragraph 2:*

*“To further substantiate that pseudogenes can be translated in vivo we interrogated the neXtprot human proteomics database (Zahn-Zabal et al. 2020). Eleven potentially-coding pseudogenes have entries in nextprot of which four, HMGB1P1, SUMO1P1, MSL3P1 and PLEKHA8P1 have matched unique peptides (Fig. S6).”*

3. The FIRRE transcript appears to have two processed pseudogenes overlapping the transcripts annotated in ENSEMBL MRCIP2P1 and also PNKDP1. Does the 2nd pseudogene PNKDP1 also have a spliced isoform and should it be added to Fig S6b?

*We have added the pseudogene PNKDP1 to Fig. S8b. It is not part of a spliced transcript, in our dataset at least.*

4. The authors mention the expression levels of the expressed pseudogenes being different than their parent genes but in Fig S8a-b the expression profile of the pseudogenes across the large exons are not uniform compared with the parent exons and is this due to potential mis- mapping of the short illumina reads?

*We saw this pattern as well. Our explanation is that reads are not uniformly distributed across pseudogene transcripts due to regions of poor mappability and 3’ bias in RNA-seq. If the alignments were caused by mis-mapping we would expect to see a high correlation between the expression of the parent and pseudogene when we instead see distinct patterns of expression.*

**Second round of review**

**Reviewer 3**

I think the authors have been diligent to address all my concerns around first version of the manuscript. The four pseudogenes with matched unique peptides is a strong indication that these pseudogenes are translated although their potential functionality is yet to identified. The additional work on examining the dN/dS of the conserved pseudogenes also contributes to the potential for their functionality. One very minor point concerning the polyadenylated pseudogenes, did the authors also identified polyadenylated signals within the pseudogene sequences?
